# Rapid pyritization in the presence of a sulfur/sulfate-reducing bacterial consortium

**DOI:** 10.1038/s41598-020-64990-6

**Published:** 2020-05-19

**Authors:** Jasmine S. Berg, Arnaud Duverger, Laure Cordier, Christel Laberty-Robert, François Guyot, Jennyfer Miot

**Affiliations:** 1 Institut de Minéralogie, Physique des Matériaux et Cosmochimie, Sorbonne Université, Muséum National d’Histoire Naturelle, CNRS UMR 7590, IRD 206 Paris, France; 2 Department of Environmental Systems Science, Institute of Biogeochemistry and Pollutant Dynamics, ETH Zurich, Zurich, Switzerland; 3 Institut de Physique du Globe de Paris, Sorbonne Paris Cité, Université Paris Diderot, UMR, CNRS 7154 Paris, France; 4 Laboratoire de Chimie de la Matière Condensée de Paris, Université Pierre et Marie Curie, Paris, France; 5 Institut Universitaire de France, Paris, France

**Keywords:** Water microbiology, Geochemistry

## Abstract

Sedimentary pyrite (FeS_2_) is commonly thought to be a product of microbial sulfate reduction and hence may preserve biosignatures. However, proof that microorganisms are involved in pyrite formation is still lacking as only metastable iron sulfides are usually obtained in laboratory cultures. Here we show the rapid formation of large pyrite spherules through the sulfidation of Fe(III)-phosphate (FP) in the presence of a consortium of sulfur- and sulfate-reducing bacteria (SRB), *Desulfovibrio* and *Sulfurospirillum*, enriched from ferruginous and phosphate-rich Lake Pavin water. In biomineralization experiments inoculated with this consortium, pyrite formation occurred within only 3 weeks, likely enhanced by the local enrichment of polysulfides around SRB cells. During this same time frame, abiotic reaction of FP with sulfide led to the formation of vivianite (Fe_3_(PO_4_)_2_·8H_2_O) and mackinawite (FeS) only. Our results suggest that rates of pyritization *vs*. vivianite formation are regulated by SRB activity at the cellular scale, which enhances phosphate release into the aqueous phase by increased efficiency of iron sulfide precipitation, and thus that these microorganisms strongly influence biological productivity and Fe, S and P cycles in the environment.

## Introduction

Pyrite (FeS_2_) is the main sedimentary sink for sulfur over geological time scales. Because the burial of (di)sulfides leaves behind oxidized products, pyrite burial exerts a major control on the oxidation state of the ocean-atmosphere system^1–3^. The ubiquitous occurrence of pyrite in marine and freshwater sediments^4–7^ makes it a keystone for the reconstruction of past biogeochemical conditions at the Earth’s surface.

There are two generally accepted pathways for pyrite formation, both starting from an FeS (*e.g*. mackinawite) precursor: the polysulfide^8,9^ pathway via which zero-valent sulfur acts as an oxidant for FeS producing FeS_2_ via the non-obligate intermediate greigite (Fe_3_S_4_)^10^, and the H_2_S pathway resulting in the formation of H_2_^8,9,11^. While sulfur- and sulfate-reducing bacteria (SRB) are the main source of sulfide (H_2_S or HS^−^) for FeS formation in sediments, their role in the formation of pyrite is still widely debated. Based on isotopic signatures (∂^56^Fe and ∂^34^S) in sedimentary pyrites, it has been suggested that pyritization can be driven by a combination of abiotic processes (diagenesis) and microbial Fe and S reduction^12,13^. However, pyrite has rarely been obtained in mixed bacterial cultures^14,15^ and even more rarely in pure cultures of SRB^16^, which instead promote the formation of metastable Fe sulfides such as amorphous FeS, mackinawite, pyrrhotite or greigite^17–20^. These minerals are usually associated with cell walls or extracellular polymeric substances (EPS) produced by bacteria^18,19^. A single study of pure cultures of *D. desulfuricans*^16^ reported the formation of pyrite and marcasite from the sulfidation of Fe(III)-oxyhydroxide (goethite) after 3 to 6 months. This suggests that the presence of Fe(III) promotes the formation of pyrite, as confirmed more recently in abiotic experiments^21,22^. Moreover, pyrite has been obtained in cultures and enrichments of sulfur-disproportionating bacteria^23,24^ containing ferrihydrite to scavenge sulfide, suggesting that intermediate redox sulfur species also play a role in pyrite formation^21,22^.

In phosphate-rich environments, pyritization competes with the precipitation of reduced iron phosphates such as vivianite (Fe_3_(PO_4_)_2_.8H_2_O) for free Fe(II), leading to associations of pyrite with phosphate minerals in both ancient and modern-day sediments^7,25,26^. During the Archean and late Proterozoic, vivianite precipitation may have exerted a strong control on the amount of phosphorus available for organic matter production and burial even in the low sulfate and euxinic paleo-ocean, thus ultimately influencing atmospheric oxygen levels^27^. The mechanism of vivianite formation has recently regained interest due to the importance of iron for the long-term retention of phosphorus under anoxic conditions, especially in modern eutrophized environments^28–30^. While much remains unknown about natural vivianite formation, it is clear that its authigenesis is governed by bulk chemical conditions such as the rate of sulfide formation relative to Fe^2+^ availability^29^. Nevertheless, the role of microorganisms in controlling/influencing the successive formation of iron sulfides and phosphates has not yet been explored.

Here we investigated the role of S-reducing microorganisms (including both sulfate and sulfur reducers) in the formation of pyrite and vivianite in enrichment cultures from ferruginous Lake Pavin water amended with amorphous Fe(III)-phosphate (FP) as an iron mineral precursor. Lake Pavin is rich in both dissolved phosphate and Fe^2+^, with additionally high amounts of particulate iron primarily composed of amorphous Fe(III)-phosphate (and a minor fraction of iron oxyhydroxides) at the chemocline. FP is gradually reduced to vivianite with depth^31^. Despite the low free sulfide (<20 µM) concentrations in the water column, it has been hypothesized that pyrite forms just below the redox boundary preserving the Fe isotope signature of dissolved Fe^2+^ there before sinking to the sediment^5^. Previous studies of Fe biomineralization in Lake Pavin have shown that microorganisms likely play a key role in the formation of Fe-bearing minerals^32^. The water column hosts a plethora of microorganisms potentially involved in Fe, S and P cycling^33,34^ and SRB in particular are more abundant and diverse than the low *in situ* sulfate and sulfide concentrations would suggest^34^. Microbial consortia retrieved from this lake are thus good candidates to evaluate the role of microorganisms in the formation of Fe sulfides and Fe phosphates in the water column, which are eventually buried and transformed within the sediment. We therefore selected an enrichment culture of Fe- and S-reducing microorganisms from Lake Pavin that were shown to produce vivianite and iron sulfides^34^ to evaluate the role of abiotic *vs*. microbially-mediated pathways in the reduction of Fe(III)-phosphate and formation of pyrite and vivianite.

## Results

### Microbial diversity

Biomineralization experiments were inoculated with a sulfur/sulfate-reducing bacterial consortium from Lake Pavin enriched on nanometric amorphous FP, sulfate and lactate. The sulfur/sulfate-reducing consortium was obtained by four successive transfers of the original enrichment dominated by *Desulfovibrio* and *Sulfurospirillum*^34^ to fresh medium. An aliquot of a 30-day-old consortium culture was used as inoculum for short-term incubations and sequenced for comparison of the bacterial diversity before and after biomineralization experiments.

**Figure 1:**
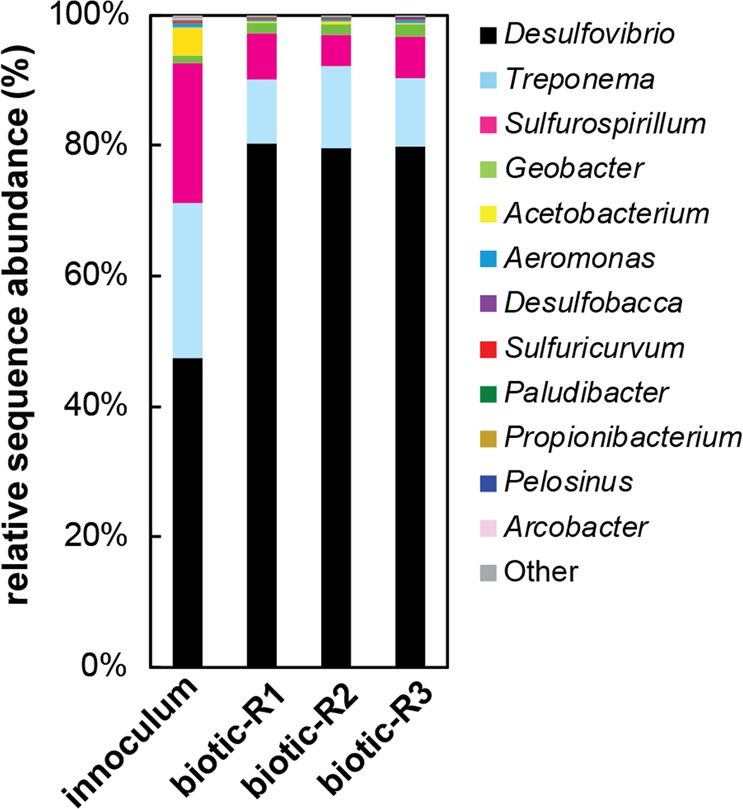
Bacterial diversity in biotic experiments from Lake Pavin. Relative sequence abundances in 16S rRNA gene amplicon libraries are shown for the inoculum and 1 month after inoculation in triplicate incubation bottles (R1, R2, R3). Only genera with greater than 0.05% abundance are shown and the remainder are included as “other”.

Based on 16S rRNA gene amplicon sequencing, the sulfur/sulfate-reducing consortium inoculum was dominated by sulfate-reducing *Desulfovibrio* (47%) and sulfur-reducing *Sulfurospirillum* (21%) (Fig. 1), which have previously been detected in the water column of the lake^34^. A few sequences belonging to other sulfur cycling bacteria such as the sulfate-reducing *Desulfobacca*, as well as the sulfur/sulfide-oxidizing *Sulfuricurvum* and *Arcobacter* were recovered, but together their relative abundance was below 0.5%. Iron-reducing bacteria (IRB) were also present but represented only a small fraction of 16S libraries. Sequences closely affiliating with the obligate Fe-reducer *Geobacter* comprised 1.1% of the initial sequenced population and facultative Fe-reducers were even scarcer. The remainder of sequences belonged mostly to fermentative genera, such as the Spirochaete *Treponema* which has been recovered from the anoxic zones of other meromictic lakes^35–37^.

At the end of the 1-month incubation in biomineralization medium containing 11 mM FP and 10 mM sulfate, the bacterial community composition exhibited remarkably little variation across triplicates (Fig. 1). The abundance of *Desulfovibrio* sequences increased to an average proportion of 80% ± 0.3%, likely in response to the high sulfate concentrations in the culture medium, whereas the abundance of *Sulfurospirillum* sequences decreased to 6% ± 1.1%. The fraction of *Geobacter* increased slightly to 1.7% ± 0.1% of final populations.

### Chemical speciation

**Figure 2:**
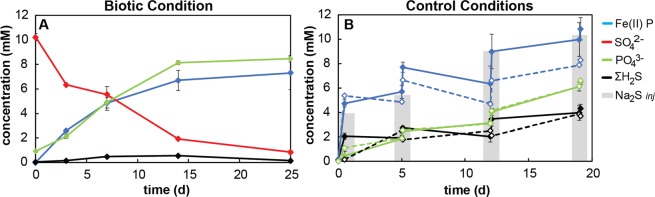
Concentrations of chemical compounds in Fe-mineralization experiments under biotic conditions (**A**) and in controls (**B**). Dotted lines in (**B**) represent sterile-filtered controls, solid lines represent killed controls, and gray bars represent the theoretical concentration of added sulfide.

Following a 10-day lag period after inoculation into the biomineralization medium of our short-term incubations, sulfate reduction to sulfide was evidenced by a rapid decrease in sulfate concentrations and the precipitation of black iron sulfides (Fig. 2A). As no significant chemical changes were observed before day 10, this time point was considered the start (t_0_) of the biotic experiment. Over an incubation period of 25 days following t_0_, the pH of the buffered medium rose only slightly, from 6.32 to 6.50 ± 0.01. During this interval, 9.2 mM (or 92%) of sulfate was consumed, with a sulfate reduction rate ranging from 0.15 to 1.2 mM per day (Fig. 2A). Interestingly, no parallel increase in sulfide was observed, with the total reactive sulfide species (H_2_S, HS^−^, S_2_^−^, S_x_^2−^, FeS) remaining below 0.54 mM throughout the experiment (Fig. 2A).

Parallel control incubations were conducted, one with sterile-filtered medium to investigate the abiotic chemical reactions between sulfide and FP (sterile control), and a second with glutaraldehyde-killed cells to differentiate the effects of cell surfaces and organic matter from true biological activities (killed control). Periodic injection of a sterile Na_2_S solution to these control incubations at concentrations mimicking biological sulfate reduction resulted in the immediate formation of black iron sulfides. The total sulfide concentration increased steadily with each Na_2_S injection but was consistently only 40–60% of the amount of sulfide added (Fig. 2B). The pH of these buffered controls rose more than in the biotic experiments, evolving from 6.32 to 7.13 ± 0.01 in the sterile controls and from 6.28 to 6.96 ± 0.01 in the killed controls.

The production/addition of sulfide can be expected to reductively dissolve Fe(III)-phosphate. Phosphate ions and Fe^2+^ were released into solution, the latter of which immediately precipitated as FeS, effectively maintaining extremely low dissolved iron concentrations (<0.6 mM) in all experiments. Fe(III) reduction was instead evidenced by an increase in the proportion of total (solid + dissolved) Fe(II) to 7.3 ± 1.4 mM in the biotic experiment (Fig. 2A). Total Fe(II) also increased to 8.3 ± 0.9 mM and 10.8 ± 0.9 mM in the sterile and killed controls, respectively (Fig. 2B). In parallel, dissolved phosphate increased to 8.46 ± 0.26 mM, 6.61 ± 0.37 mM and 6.49 ± 0.22 mM in the biotic experiment, sterile control and killed control, respectively, after 3 weeks (Fig. 2A,B).

### Evolution of iron minerals

**Figure 3:**
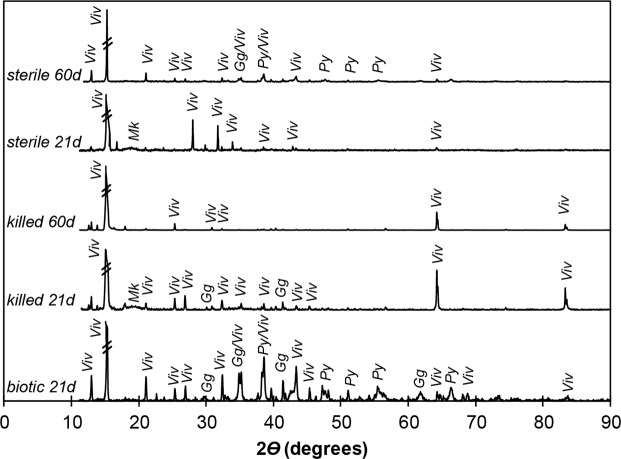
X-ray diffractograms of mineral precipitates in biotic experiments with the sulfur/sulfate-reducing consortium from Lake Pavin after 21 days of incubation and killed and sterile control experiments with added Na_2_S after 21 and 60 days of incubation. *Viv* = vivianite, *Mk* = mackinawite, *Py* = pyrite, *Gg* = greigite.

The bulk mineralogy of solid-phase precipitates formed in the biotic experiment and in the sterile and killed controls was analyzed by XRD after 3 weeks of incubation (Fig. 3). The reduced iron-phosphate mineral vivianite (Fe_3_(PO_4_)_2_.8H_2_O) was detected in all experimental conditions. In addition, a wide peak around 20° of *2θ* angle (Co Kα) attributed to low-ordered mackinawite was apparent in both the sterile and killed controls (Fig. 3) but not in the biotic experiment. It is possible that some mackinawite also precipitated in the biotic condition but in amounts below the detection limit of XRD or in a very poorly crystalline form. Over this timescale, the mixed-valence iron mineral greigite (Fe_3_S_4_) as well as pyrite were detected only in the biotic experiment with the active sulfur/sulfate-reducing consortium. In contrast, greigite and pyrite were only observed after 2 months in the sterile control, and not in the killed control (Fig. 3).

For comparison, the mineralogy of the 75-day old original enrichment culture (inoculum) was investigated by Fe K-edge EXAFS revealing the coexistence of vivianite, mackinawite, greigite and pyrite, with 10% of Fe remaining as original Fe(III)-phosphate (Supplementary Fig. SI1 and Table SI1).

### Cell-mineral associations

**Figure 4:**
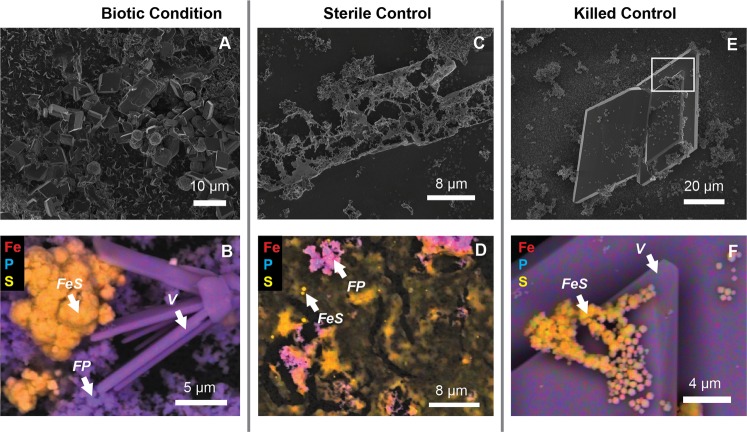
Electron microscope analyses of mineral precipitates in biotic and control experiments. SEM images (top panels) and EDX maps (bottom panels) reveal the morphology and composition, respectively, of precipitates from biotic experiments with the sulfate-reducing Lake Pavin enrichment culture (**A**,**B**), the sterile control (**C**,**D**) and the killed control (**E**,**F**). White box in (**E**) shows region analyzed in (**F**). Fe-S and Fe-P associations appear in orange and purple, respectively. *V* = vivianite, *FP* = Fe-phosphate, *FeS* = FeS beads.

**Figure 5:**
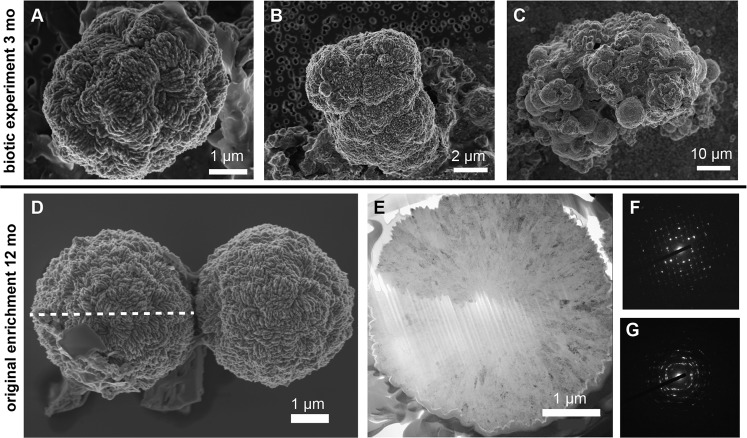
Pyrite spherules from the biotic experiment and original sulfate-reducing Lake Pavin enrichment culture. (**A**–**C**) SEM images revealing the growth and aggregation of pyrite spherule in the biotic experiment after 3 months of incubation. (**D**–**G**) Spherical mineral precipitates from the original enrichment culture after 12 months. (**E**) Cross-section of spherule imaged by SEM in (**D**) was obtained by focused ion beam cutting along the dotted axis. Electron diffraction patterns reveal polycrystalline domains of greigite (**F**) and pyrite (**G**).

The evolution of the textures and compositions of mineral precipitates formed in the biotic experiment and in sterile and killed controls were monitored by SEM-EDX (Fig. 4). After 25 days of incubation, precipitates from biotic incubations contained abundant angular Fe- and P-bearing crystals, which is consistent with the morphology and chemical composition of vivianite (Fig. 4A,B). Rounded Fe- and S-rich beads around 1 µm in diameter were also observed and appeared to grow and aggregate over time (Fig. 5A–C), evolving into spherules measuring 2 to 5 µm in diameter after 3 months.

In the sterile and killed controls, vivianite crystals were present (Fig. 4E,F) along with FP nanoparticles (Fig. 4D) and thin films composed of Fe and S (Fig. 4C,D) after 25 days of incubation. STEM-EDX analysis of these sterile and killed controls confirmed the precipitation of iron sulfides with a chemical composition and crystalline structure consistent with mackinawite (Supplementary Fig. SI2), in agreement with XRD data (Fig. 3). After 2 months, spherules of iron sulfide, measuring circa 500 nm in diameter were observed in the sterile control and were even smaller (and not detected by XRD, Fig. 3) in the killed control (Supplementary Fig. SI3).

**Figure 6:**
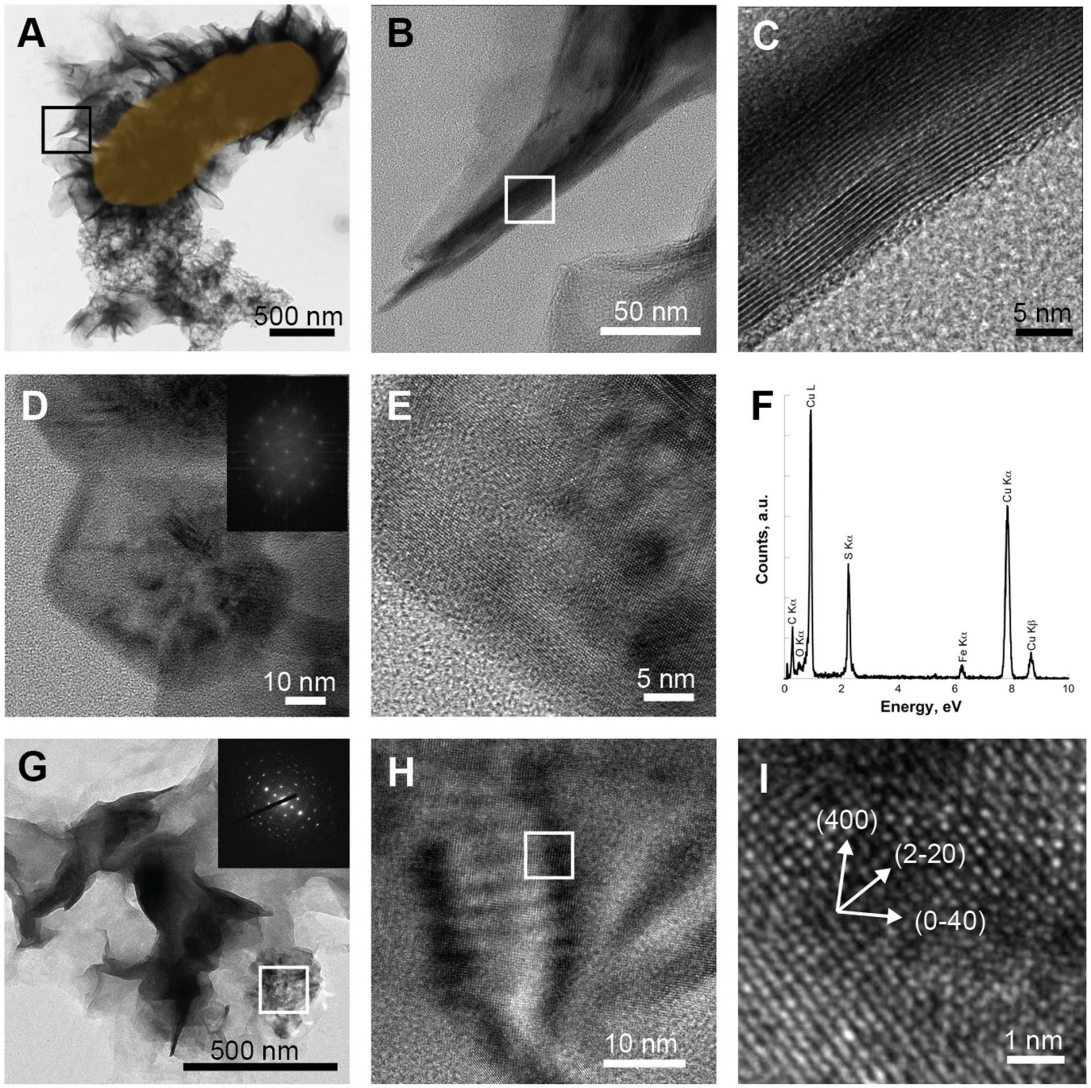
HRTEM analyses of mineral precipitates in the original enrichment culture in the presence of Fe(III)-phosphate and lactate, after 35 days (**D**–**F**) or 50 days (**A**–**C** and **G**–**I**). (**A**–**C**) HRTEM images of a mackinawite-encrusted microbial cell in (**A**) shown in orange, with interplanar distances of 5.1 Å consistent with *d(hkl)* = *d(001)* of mackinawite (**B**,**C**). Inset in (**D**) shows the Fourier Transform signal of an image of elemental sulfur nanoparticles. (**E**) is a magnified view of the same particle and (**F**) gives its elemental composition obtained by EDX. (**G**–**I**) TEM images of greigite, with electron diffraction pattern and HRTEM ((00–1) zone axis).

**Figure 7:**
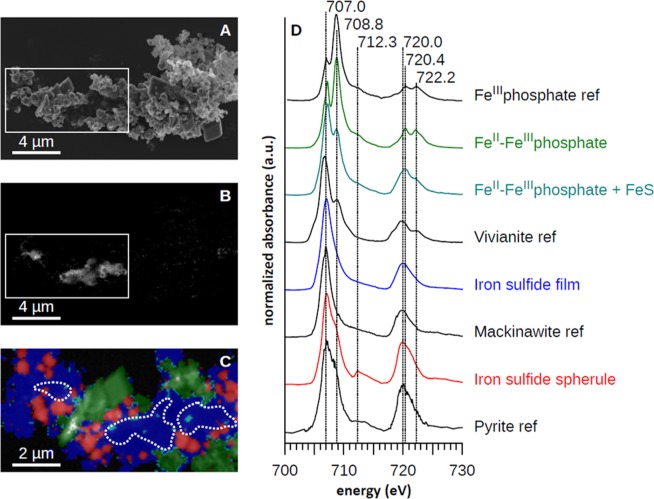
STXM analysis of cell-mineral associations in the original enrichment culture after 50 days of incubation. (**A**) SEM image of the area analysed by STXM, (**B**) STXM organic carbon map obtained by subtracting the image at 280 eV from the image at 288.2 eV. (**C**) STXM composite map with three components: Fe(II)-Fe(III) phosphate (red), Fe(II)-sulfide (blue) and Fe(II)-phosphate (green). (**D**) Normalized Fe L_2,3_-edges XANES spectra of the three components and of reference amorphous Fe(III) phosphate, mackinawite and vivianite.

Microbe-mineral associations were also closely examined in the 50-day old original Lake Pavin enrichment culture using TEM (Fig. 6). Bacteria appeared to be encrusted in aggregated platelets of FeS. This mineral phase exhibited a local nanocrystalline organization consistent with mackinawite (Fig. 6A–C). Nanoparticles of hexagonal elemental sulfur were also identified based on their chemical composition and electron diffraction patterns (Fig. 6D–F). Greigite particles (Fig. 6G–I) were detected in the proximity of mackinawite/amorphous FeS thin films. Further analyses by STXM at the Fe L_2,3_-edges and C K-edge revealed protein-rich areas surrounded by Fe-bearing minerals exhibiting the same NEXAFS spectrum as reference mackinawite (Fig. 7), which suggests the presence of bacteria^38,39^ surrounded by mackinawite. This is in accordance with the detection of mackinawite by EXAFS at the Fe K-edge in this sample (Supplementary Fig. SI1, Table SI1). Nanoparticles of pyrite, as well as mixed Fe^II^-Fe^III^ phosphates, were also found to co-exist in the vicinity of the cells (Fig. 7).

After significant aging (12 months) of this original enrichment culture, numerous Fe- and S-rich spherules measuring 4–5 µm in diameter were observed by SEM (Fig. 5D, Supplementary Fig. SI4). Thin foils of these large spherules were analyzed by TEM after FIB milling (Fig. 5E), revealing an internal elemental composition of S and Fe varying in ratios (S:Fe) from 1.5 to 2, which falls between the elemental ratios of greigite (S:Fe = 1.3) and pyrite (S:Fe = 2). Electron diffraction confirmed that spherules consisted of polycrystalline pyrite (Fig. 5F) hosting small greigite domains (Fig. 5G).

## Discussion

Based on the high (~10%) relative abundance of SRB sequences in 16S rRNA gene sequence libraries from the chemocline and their dominance in enrichment cultures, SRB have previously been suggested to play an important role in the redox transformation of iron in the sulfate-poor waters of Lake Pavin^34^. In our enrichment from this lake, the dominant populations of *Desulfovibrio* and *Sulfurospirillum* were likely responsible for sulfide production driving indirect Fe(III)-phosphate reduction, while the minor proportion of iron-reducing *Geobacter* present may have played a minor role in direct reduction of Fe(III) minerals^40,41^ (Figs. 1 and 2). Fermentative bacteria may also have contributed to Fe(III) reduction indirectly, using iron as a sink for excess reducing equivalents. This may explain the abundance of *Treponema* sequences, as at least one species from this genus has thus far been reported to reduce poorly crystalline Fe(III) to vivianite^42^. Nonetheless, the inverse relationship between sulfate reduction and Fe(II) production (Fig. 2A) suggests the predominant mechanism of Fe(III)-phosphate reduction at play was reductive dissolution by sulfide:

1 $${{\rm{HS}}}^{-}+2\,{{\rm{Fe}}}^{{\rm{III}}}{{\rm{PO}}}_{4}+{{\rm{3H}}}^{+}\to {{\rm{S}}}^{0}+2\,{{\rm{Fe}}}^{2+}+2\,{{\rm{H}}}_{2}{{{\rm{PO}}}_{4}}^{-}$$

This reaction implies that one mole of sulfide can reduce two moles of Fe^III^PO_4_ and release two moles of Fe^2+^. However, the production of polysulfides (S_x_^2−^) rather than elemental sulfur would shift the stoichiometry towards an equimolar reaction of sulfide and Fe(III)-phosphate as has been shown for abiotic surface-mediated reaction of HS^−^ with Fe-oxyhydroxides^21,22^.

In sterile and killed controls, sulfide addition indeed promoted Fe(III)-phosphate reduction via an equimolar reaction of sulfide with Fe(III) (Fig. 2B). Both controls were very similar, with a maximum of 4 mM sulfide (mostly as FeS) measured after reaction of 10 mM Na_2_S with Fe(III)-phosphate over 3 weeks (Fig. 2B). This suggests that remaining sulfide was converted into a pool of intermediate redox sulfur species (most likely polysulfides).

In the biotic experiment, the reduction of 9 mM sulfate also drove an equimolar reaction of sulfide with Fe(III) (Fig. 2A), but the concentration of reactive sulfide remained extremely low. This suggests that either (1) the dimethyl-p-phenylene-diamine used to complex sulfide did not react with sulfide bound in mineral form, and/or (2) sulfur species accumulated as a large pool of intermediate sulfur compounds (e.g. elemental sulfur, polysulfides). The first hypothesis is supported by the presence of crystalline Fe sulfides not detectable by the methylene blue method (i.e. greigite, pyrite, Fig. 3). The second hypothesis was not directly evaluated (no measurement of intermediate sulfur compounds) but is supported by several observations. First, intermediate sulfur compounds may have resulted from both abiotic Fe(III)-reduction by sulfide (Eq. 1) and from sulfide oxidation by *Sulfuricurvum* and *Arcobacter* (Fig. 1) possibly stimulated by low amounts of nitrate in the biomineralization medium prepared from sterile-filtered Lake Pavin water^32^. Second, intermediate sulfur compounds may have supported the growth of *Sulfurospirillum* that are incapable of reducing ferric iron or sulfate but instead utilize elemental sulfur, polysulfides, and thiosulfate as electron acceptors^43^ (Fig. 1).

More or less crystalline iron sulfide phases may originate from the reaction of excess sulfide added as Na_2_S in the sterile and killed controls or produced by SRB in biotic experiments with Fe(III)-phosphate, as follows:

2 $$3{{\rm{HS}}}^{-}+2{{\rm{Fe}}}^{{\rm{III}}}{{\rm{PO}}}_{4}+{{\rm{H}}}^{+}\to 2{{\rm{Fe}}}^{{\rm{II}}}{\rm{S}}+{{\rm{S}}}^{0}+2{{\rm{H}}}_{2}{{{\rm{PO}}}_{4}}^{-}$$

After 3 weeks, mackinawite was indeed detected by TEM and XRD in the sterile and killed controls (Figs. 3, SI2). Rounded nanoparticles associated with FeS films were interpreted to be elemental sulfur rather than pyrite based on EDX analyses and electron diffraction patterns (Supplementary Fig. SI2). Over longer time scales (2 months; Supplementary Fig. SI3), these assemblages evolved into greigite and pyrite in the sterile control, which is consistent with previously described pyrite formation pathways^11,44–46^:

3 $$3{\rm{FeS}}+{{\rm{S}}}^{0}\to {{\rm{Fe}}}_{3}{{\rm{S}}}_{4({\rm{greigite}})}$$

4 $${{\rm{Fe}}}_{3}{{\rm{S}}}_{4({\rm{greigite}})}+2{{\rm{S}}}^{0}\to 3{{\rm{FeS}}}_{2({\rm{pyrite}})}$$

Nevertheless, pyrite formation was not observed at any time point in the killed control, suggesting that cell surfaces are not mandatory for pyrite formation. Although the glutaraldehyde-treated cells were thoroughly rinsed, we cannot exclude that trace amounts of aldehydes persisted in our experiments which could have oriented the transformation of mackinawite to greigite and inhibited the formation of pyrite^47^.

In the biotic experiments, no mackinawite could be detected by XRD after 3 weeks (Fig. 3). This suggests that mackinawite was present at very low levels in this sample and may have been only a transient phase. In addition, sulfide precipitated in the form of greigite and of pyrite spherules (Fig. 3) that reached 5 µm in diameter after 3 months. In contrast, well-crystallized mackinawite was associated with greigite and pyrite in the 50–75 day old original inoculum as shown by XRD^34^, TEM (Fig. 6A–C), STXM (Fig. 7) and EXAFS (Supplementary Fig. SI1, Table SI1). The pyrite particles evolved into spherules of 5 to 10 µm in diameter after 12 months (Supplementary Fig. SI4).

The difference in mackinawite textures under abiotic and biotic (Fig. 4, Supplementary Fig. SI2 & 3) conditions, including the encrustation of microbial cells in FeS (Fig. 6A–C), suggests that SRB play a role in the precipitation of this mineral. In fact, it is known that SRB are involved in mackinawite formation via enhancement of local sulfide concentrations and by acting as nucleation templates for crystal growth^19^. Similarly, the nucleation of greigite nanoparticles at cell surfaces (Fig. 6G–I) is in accordance with previous reports of greigite formation in SRB enrichments and pure cultures^19,48–52^. This mineral is indeed reported to form more rapidly in the presence of live SRB cells^19^. Under reducing conditions, this transformation may be limited by the source of oxidants for Fe^2+^ required for the formation of this mixed-valence mineral (Fe^II^Fe^III^_2_S_4_). In our biotic experiments, the transformation was extremely rapid (<1 month) compared to that reported from pure SRB cultures (>5 months) in the presence of Fe^2+ 19^. This difference is likely due to the presence of ferric iron in our experiments, which could generate abundant S^0^ (Fig. 6D–F) and/or polysulfides upon reaction with sulfide (Eqs. 1 & 2), which might enhance the formation of greigite and then pyrite, following Eqs. (3) and (4). It has been shown that under abiotic conditions and in the presence of Fe(III)-oxyhydroxides (goethite or lepidocrocite), quasi-instantaneous precipitation of FeS competes with the formation of pyrite^22^. The precipitation of pyrite is favored by (a) a globally high Fe(III)/S(-II) ratio (>2), (b) the presence of available sites at ferric oxy-hydroxide surface to form a >Fe(II)S(-II) precursor, (c) the capacity of the iron oxide to transfer electrons from its surface towards its inner structure and (d) the attainment of a local critical supersaturation with respect to FeS_2_ via FeS dissolution^21,22,53^. The high specific surface area of the amorphous Fe(III)-phosphate (nanoparticles)^54^ likely ensured both a high Fe(III)/S(-II) ratio and the availability of reaction sites at the FP surface favoring greigite and pyrite formation. Furthermore, the local delivery of polysulfides in the vicinity of SRB cells would have accelerated greigite and pyrite formation in the biotic condition. The formation of pyrite was much more rapid in the biotic experiments compared to the original enrichment, most likely because successive transfers to fresh medium maintained cells in active growth phase with high metabolic rates, meaning higher rates of polysulfide release. The formation of pyrite in pure cultures or enrichments of SRB has rarely been reported^14,15^ and never within such rapid time scales as 3 weeks. Moreover, pyritization occurred despite the potential inhibitory effect^47,55^ of rather large amounts of organic acids present in the cultures (resulting from incomplete lactate oxidation).

In the sedimentary record, pyrite occurs frequently in the form of framboids, *i.e*. sub-spherical to spherical structures 1–10 µm in diameter, composed of more or less organized assemblages of microcrystals^56^. The pathways of pyrite framboid formation and their potential biogenic origin remain subject to debate^57^. Although they did not display the same texture as pyrite framboids described in the literature^58,59^, spherules that formed in our biotic experiments and original enrichment shared some features with framboids: (a) they were in the same size range, (b) they were polycrystalline suggesting growth from multiple nucleation sites, and thus a high nucleation rate. In addition, spherules were composed of adjacent nanodomains of pyrite and greigite suggesting a syngenetic relationship between these two minerals. These results provide evidence of pyrite formation through the conversion of greigite (following Eq. 4) in the presence of S(0) and polysulfides^10^ and enhanced by SRB activity. The possibility that these spherules may be transient precursors that are transformed to pyrite framboids during early diagenesis, in particular upon temperature/pressure increase, would deserve further investigation.

There is a long-lasting debate surrounding the role of organic matter in pyrite framboid formation^60–63^. Whereas framboids have been successfully synthesized under purely abiotic conditions (in the absence of organic molecules)^64^, pyrite framboids have often been observed in association with organics such as humic substances, EPS or extracellular DNA^61,65,66^ that may provide nucleation sites for pyrite (or precursors) precipitation. However, traces of organic matter were not observed within the pyrite spherules formed in the original enrichment (Fig. 5) suggesting that the presence of microbial cells/organic matter was not mandatory for pyrite nucleation and growth. This is further supported by the formation of small spheres of pyrite (500 nm in diameter) in the sterile control and of even smaller iron sulfide spheres not detectable by XRD (probably due to low crystallization or low abundance) in the killed control (Fig. 3; Supplementary Fig. SI3). As suggested in previous studies e.g.^15^, it appears that cell surfaces are not mandatory to promote pyrite formation. Most likely, it is the local production of sulfide by SRB in the presence of Fe(III) which resulted in locally high levels of polysulfides and promoted rapid (3 weeks) greigite and pyrite formation. Whether some bacteria could retrieve energy from these S-mediated redox reactions, as discussed in^15^ remains to be explored in our system.

Under both biotic and abiotic conditions, part of the Fe(II) was actually precipitated in the form of vivianite. This mineral is increasingly reported as a major sink for phosphorus in both freshwater^29,67^ and marine environments^7,26,68^. Vivianite and pyrite have been shown to co-exist at or across the sulfate-methane transition zone in marine sediments^7,69^. Generally, vivianite is reported to form only if iron availability exceeds dissolved sulfide concentrations, so that dissolved iron persists after iron sulfide precipitation^29,70^. Interestingly, thermodynamics predict that the following equilibria are reached successively:

5 $$3{{\rm{HS}}}^{-}+8{{\rm{H}}}_{2}{\rm{O}}+6{{\rm{Fe}}}^{{\rm{III}}}{{\rm{PO}}}_{4}+{{\rm{H}}}^{+}\to 2{{{\rm{Fe}}}_{3}}^{{\rm{II}}}{({{\rm{PO}}}_{4})}_{2}\times 8{{\rm{H}}}_{2}{\rm{O}}+2{{\rm{H}}}_{2}{{{\rm{PO}}}_{4}}^{-}+3{{\rm{S}}}^{0}$$

6 $$2{{\rm{HS}}}^{-}+{{\rm{Fe}}}^{{\rm{III}}}{{\rm{PO}}}_{4}+{{\rm{H}}}^{+}\to {{\rm{Fe}}}^{{\rm{II}}}{{\rm{S}}}_{2}+{{\rm{H}}}_{2}{{{\rm{PO}}}_{4}}^{-}+1/2\,{{\rm{H}}}_{2}$$

Based on simple thermodynamic modeling of our systems using the CHESS geochemical software^71^ (Supplementary Fig. SI5), sulfide always promotes FP dissolution and vivianite precipitation below sulfide concentrations of 2.5 mM. Whereas under low sulfide concentrations (<2.5 mM), typically reached at the beginning of our experiments, vivianite is the only thermodynamically stable mineral predicted, it systematically co-exists with iron sulfides (FeS or FeS_2_) at higher sulfide concentrations. Simulations of higher sulfide concentrations (above 15 mM) that may be reached in the vicinity of SRB cells predict complete vivianite dissolution and phosphate release into solution. This is consistent with the higher dissolved phosphate concentrations measured in our biotic experiments (up to 8.46 ± 0.26 mM) compared to the controls (6.61 ± 0.37 mM and 6.49 ± 0.22 mM in the sterile and killed controls, respectively) (Fig. 2). Phosphate recycling controlled by the competition between vivianite and Fe sulfide formation has been proposed to regulate phosphate levels under the low sulfate euxinic conditions of Lake Cadagno and in past environments from the early Archean to late Proterozoic, with important consequences on the control of biological productivity and organic matter burial^27^. Our study suggests that local chemistry in the surroundings of SRB cells is an important parameter contributing to these processes. These drastic changes in the local environment of crystallization could even alter the isotopic signatures preserved in pyrites, which deserves further investigation. Finally, while most of the mineral transformations described here are driven by abiotic reactions with sulfide (Eqs. 1 and 2), SRB appear to play an essential role in the kinetics of pyrite formation and in the regulation of Fe, S, and P biogeochemical cycles.

## Methods

### Culture medium preparation

To ensure anoxic conditions, all sample handling and experiments were performed in a JACOMEX® glove box maintained under Ar atmosphere (Alphagaz 1, Air Liquide, [O_2_] < 10 ppm). All vitamin, trace element, nutrient and buffer solutions were prepared with O_2_-free bi-distilled water (ddH_2_O) obtained by argon bubbling (Alphagaz 1, Air Liquide) for 45 min at 80 °C. Solutions were sterilized by autoclaving except for the vitamin solution which was filter-sterilized (0.2 µm). Amorphous nanometer-sized Fe(III)-phosphate was synthesized as described in the literature^34,54^.

The original enrichment was set up using Lake Pavin water with added amorphous Fe(III)-phosphate (11 mM), sulfate (10 mM), and Na-lactate (20 mM) and buffered with 50 mM of 2-(N-morpholino)ethanesulfonic acid (MES), pH 6.5^34^. The medium was also supplemented with 1 ml·l^−^¹ each of vitamin solution^72^, trace element solution^73^, and selenite/tungstate solution^74^. The sulfur/sulfate-reducing consortium used to inoculate biomineralization experiments was obtained by transferring 1 ml of the enrichment culture into 40 ml fresh medium prepared the same way, except that lake water was filter-sterilized (0.2 µm), every 3 months for a total of four successive transfers. The remainder of the original culture was further incubated for up to 12 months, without any substrate additions, in order to monitor the long-term evolution of the organic matter and mineral assemblies.

### Short-term incubations

To monitor water chemistry during sulfate and Fe(III) reduction, three short-term incubation experiments were performed under anoxic conditions: one biotic (=biomineralization) experiment, and two controls (a sterile-filtered control and a killed control). For each condition, incubations were performed in triplicate in 60 ml serum bottles filled with 40 ml of buffered medium and sealed with butyl rubber stoppers.

In biotic experiments, medium (supplemented with 20 mM lactate, 10 mM sulfate, and 11 mM Fe(III)-phosphate) was inoculated with 1 ml of the sulfur/sulfate-reducing consortium. The abiotic sterile-filtered control was prepared with filter-sterilized (0.2 µm) Lake Pavin medium (supplemented with 11 mM Fe(III)-phosphate) to which sterile Na_2_S solution was periodically injected at concentrations mimicking the sulfate reduction rates measured in biotic experiments.

A killed control was also prepared to evaluate the role of microbial cell surfaces or organic material in nucleating reduced iron mineral phases. Approximately 30 ml of a culture of *Desulfovibrio desulfuricans* (DSM 642, DSMZ, Germany) in stationary phase was centrifuged (6500 *g*, 10 min), and the pellet re-suspended in an anoxic solution of 4% glutaraldehyde in ddH_2_O. After 24 h of incubation at room temperature, cells were pelleted by centrifugation (6500 *g*, 10 min) and rinsed in ddH_2_O three times. After the final centrifugation, the pellet was resuspended in 40 ml of sterile Lake Pavin medium (supplemented with 11 mM Fe(III)-phosphate) to which sterile Na_2_S solution was periodically injected at concentrations mimicking the sulfate reduction rates measured in the biotic experiments.

### Chemical analyses

Samples for measurements of total and dissolved (0.2 µm-filtered) compounds in the controls and the biotic experiments were taken from serum vials using sterile syringes and needles in the anaerobic chamber. Sulfate samples were immediately sterile-filtered to prevent biological oxidation of sulfide to sulfate and diluted in ddH_2_O for analysis on an ion chromatograph (Dionex DX-600 IC System) at the Laboratory of Water Geochemistry, IPGP. Dissolved phosphate was quantified using a phosphate assay kit (Sigma-Aldrich) based on the colorimetric malachite green method. Sulfide samples were immediately fixed with zinc acetate solution (5% final concentration) and quantified via the photometric method of Cline^75^. Total and dissolved (0.2 µm-filtered) iron samples were mixed with HCl (0.5 M final concentration) and stored in the anaerobic chamber until analysis of ferrous and total iron using the ferrozine method^76^. The pH was monitored on separate liquid samples with a Sensorex© pH electrode inside the anaerobic chamber.

### Sample preparation for mineralogical analyses

Solid mineral precipitates from the original sulfate-reducing enrichment culture (after 50 days to 12 months), the biotic experiments and the controls were processed for mineralogical analyses. For X-Ray Diffraction (XRD) and transmission electron microscopy (TEM), 1–10 mL of suspended particles were pelleted by centrifugation (6500 *g*, 10 min) and rinsed three times with degassed ddH_2_O. Precipitates were resuspended in anoxic ddH_2_O, and a subsample of suspension (~3 µL) was deposited either on 200-mesh Lacey carbon Cu TEM grids (Agar) for TEM analyses, or on silicon nitride windows (Norcada, Canada) for STXM analyses. The remainder was deposited onto a silicon wafer for XRD analysis. All preparations were allowed to dry completely under Ar atmosphere before storing in gas-tight aluminum bags until further analysis. For scanning electron microcopy (SEM), 100 µL of sample was diluted in 20 mL of O_2_-free ddH_2_O and filtered onto a polycarbonate GTTP 0.2 µm filter (Merck Millipore, Darmstadt, Germany). Filters were mounted onto adhesive carbon stubs and sputter-coated with a thin layer of carbon before analysis.

### X-Ray diffraction (XRD)

Bulk mineralogy of crystalline solid phases was determined by XRD. Prepared samples were mounted in a custom-designed anoxic analysis chamber equipped with a Kapton tape window for measurement on a PANalytical X’Pert Pro MPD diffractometer in Bragg-Brentano configuration. Diffraction patterns were recorded using Co Kα radiation at 40 kV and 40 mA, in the 2θ range of 10 to 90° with a 2θ step of 0.017°. Five scans of 50 min each were recorded per sample and then integrated and analyzed using the PANalytical X’Pert Highscore software.

### Scanning electron microscopy (SEM)

A Zeiss Ultra 55 SEM equipped with a field emission gun (FEG) and a Brucker EDX Quantax detector (Brucker Corporation, Houston, TX, USA) was used for morphological and elemental characterization of mineral precipitates. Imaging was performed in secondary electron mode (In Lens detector) at 2 kV and a working distance of 2–3 mm. Energy dispersive X-ray spectrometry (EDX) analyses were performed at 15 kV and a working distance of 7.5 mm in backscattered electron mode (SE2 detector).

### Focused ion beam (FIB) milling

Focused ion beam (FIB) milling was performed using the FEI STRATA DB 235 FIB system operating at the IEMN (Lille, France) to prepare electron-transparent 80-nm thick sections of pyrite spherules from the enrichments. Milling was performed on samples deposited on a clean silicon wafer without any previous embedding at low Ga-ion currents to prevent formation of artefacts^77,78^.

### Transmission electron microscopy (TEM)

Samples deposited on Cu-grids and FIB sections were analysed by scanning transmission electron microscopy (STEM) and (high resolution) transmission electron microscopy ((HR)TEM) using the 200 kV field emission gun (FEG) JEOL2100F microscope operating at the IMPMC (Paris, France). STEM observations were performed in high-angle annular dark field (HAADF) mode and Energy Dispersive X-ray (EDX) maps were recorded. Selected-area electron diffraction (SAED) patterns were measured on areas of interest.

### Scanning transmission X-ray microscopy (STXM)

STXM analyses were performed at the HERMES beamline at SOLEIL (Saint Aubin, France) following procedures described previously^79,80^. Energy calibration was accomplished using the well-resolved 3p Rydberg peak at 294.96 eV of gaseous CO_2_ for the C K-edge and the L_3_ peak of hematite at 708.5 eV for the Fe L_2,3_-edges. First, image mapstacks for C were recorded at 288.2 and 280 eV according to published procedures to avoid irradiation damage^81^. Image stacks were then collected at the Fe L_2,3_-edges from 690 to 740 eV and Fe L_2,3_-edges NEXAFS spectra were normalized following the procedure by Bourdelle *et al*.^82^. Data were processed using the aXis2000 software^83^ following previously published methods^80^. In particular, protein maps were obtained by subtracting the image at 288.2 eV (maximum of protein absorbance) from the image at 280 eV (below the C K-edge).

### Microbial diversity analysis

We analyzed the microbial diversity of the biotic experiments at two time points: first in the inoculum used for the biotic experiments, then 1 month after inoculation in each of three triplicate incubation bottles. Using a sterile syringe, 2–5 ml was sampled from each culture and bacterial cells were concentrated onto a 0.2 µm polycarbonate filter (Millipore) mounted in an autoclaved Swinnex filter holder before freezing at −20 °C until further processing.

Bacterial DNA was extracted using the ZR Fecal DNA Kit (Zymo Research) according to the manufacturer’s protocol, and quantified fluorometrically at 260 nm using the Qubit dsDNA HS Assay KIT (Invitrogen). The extracts were frozen at −20 °C until shipping on dry ice to MrDNA (Shallowater, Texas) for sequencing. Barcoded amplicon sequencing targeting the hypervariable V3-V4 region (primers 341 F and 785 R) was performed by MR DNA using bTEFAP® technology. In summary, a single-step 30 cycle PCR using HotStarTaq Plus Master Mix Kit (Qiagen, Valencia, CA) was performed under the following conditions: 94 °C for 3 minutes, followed by 28 cycles at 94 °C for 30 seconds; 53 °C for 40 seconds and 72 °C for 1 minute; with a final elongation step at 72 °C for 5 minutes. Successful amplification was verified by gel electrophoresis. Resulting amplicon products from different samples were mixed in equal concentrations and purified using Agencourt Ampure beads (Agencourt Bioscience Corporation, MA, USA) and sequenced by Illumina MiSeq (Illumina, USA) paired end (2 × 300 bp) sequencing.

Sequence data was processed using a proprietary analysis pipeline (www.mrdnalab.com, MR DNA, Shallowater, TX). In short, paired end reads were merged and barcode and primers sequences were trimmed. Short sequences <150 bp, sequences with ambiguous base calls, and sequences with homopolymer runs exceeding 6 bp were removed. The remaining reads were then denoised, and clustered at 97% sequence similarity. Singleton sequences and chimeras were excluded from analyses. Taxonomic assignment was based on a local nucleotide BLAST^84^ search against a curated database derived from GreenGenes, RDPII and NCBI. Sequences were submitted to ENA under the study accession number (PRJEB29915/ERP112273).

## Supplementary information

Supplementary Information.

## Data Availability

All data generated or analysed during this study are included in this published article (and its Supplementary Information files).
