# Supplementary material for: Rapid pyritization in the presence of a sulfur/sulfate-reducing bacterial consortium
Source: Sci Rep. 2020 May 19;10:8264. doi: 10.1038/s41598-020-64990-6 (PMC7237684; doi:10.1038/s41598-020-64990-6)
Supplement: Supplementary file 1 — Supplementary Information. [file 41598_2020_64990_MOESM1_ESM.pdf]

Rapid pyritization in the presence of a sulfur/sulfate-reducing bacterial consortium

Jasmine S. Berg[1,2*], Arnaud Duverger[1], Laure Cordier[3], Christel Laberty-Robert[4], François Guyot[1,5], Jennyfer Miot[1]

[1]Institut de Minéralogie, Physique des Matériaux et Cosmochimie, Sorbonne Université, Muséum National d'Histoire Naturelle, CNRS UMR 7590, IRD 206, Paris, France

[2]Department of Environmental Systems Science, Institute of Biogeochemistry and Pollutant Dynamics, ETH Zurich, Zurich, Switzerland

[3]Institut de Physique du Globe de Paris, Sorbonne Paris Cité, Univ. Paris Diderot, UMR CNRS 7154, Paris, France

[4]Laboratoire de Chimie de la Matière Condensée de Paris, Université Pierre et Marie Curie, Paris, France

[5]Institut Universitaire de France

*Corresponding Author

E-mail: jasmine.berg@usys.ethz.ch (JSB)

METHODS

Preparation of reference model compounds for X-ray absorption spectroscopy at the Fe K-edge.

A large set of model compounds for XAS and STXM analyses were synthesized, including pyrite (following the procedure by [1,2]), greigite [2,3], mackinawite [4], vivianite [5], amorphous Fe(III)-phosphate (FP) [6]. The greigite reference was contaminated with residual mackinawite (proportion not determined). Thus, we were only able to give a proportion of the greigite + mackinawite mixture in Table 1.

X-ray absorption spectroscopy at the Fe K-edge.

Vacuum dried samples (references and precipitates from the 75-day old original enrichment were gently ground in an agate mortar in an anaerobic glovebox, mixed with an appropriate amount of cellulose to achieve an absorption edge height ($\Delta\mu x$) as close to 1 as possible, and compressed into pellets sealed with Kapton tape. Fe K-edge XAS spectra were collected at 77 K (liquid N_2-cryostat) in transmission mode at the XAFS beamline (ELETTRA, Italy) using a Si(111) double-crystal monochromator. The energy was calibrated by setting the first inflection point of an Fe foil K-edge recorded in double-transmission set up to 7112 eV. Spectra were merged and normalized using the software Athena[7] and Extended X-ray Absorption Fine Structure (EXAFS) data were extracted using the program XAFS [8]. X-ray Absorption Near Edge Structure (XANES) and k_3-weighted EXAFS spectra (2.5–15 Å^{-1} range) were analyzed using a Linear Combination Fit (LCF) (as described in [9,10]) using the spectra from the synthesized reference compounds. LCF components with contributions < 5% were considered as not significant.

FIGURES

Figure SI1| Fe K-edge EXAFS analysis of the original enrichment culture from 10 m below the oxycline, performed in the presence of amorphous Fe(III)-phosphate, lactate and sulfate, after 75 days of incubation. A: XANES, B: k3χ(k) and C: Fourier Transform of the EXAFS signal. Black: data; red: fit.

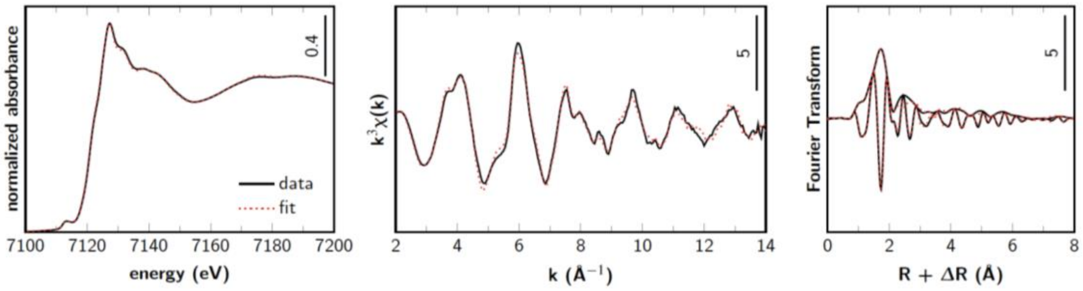

Figure SI2| STEM images and parallel EDX maps of mineral precipitates in the abiotic killed control (a,b) and sterile-filtered control (c,d) experiments after 3 weeks of incubation. The white box in (c) shows region analyzed in (d).

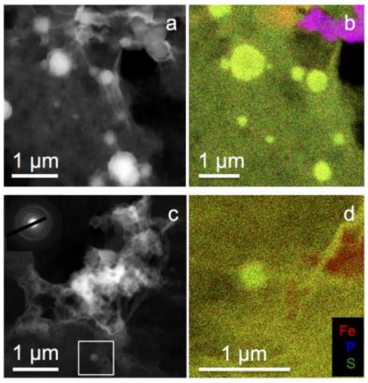

Figure SI3| SEM images and parallel EDX maps showing well-crystallized vivianite and FeS spherules in abiotic killed control (a,b) and sterile-filtered control (c,d) experiments after 2 months of incubation. (e) The upper and lower EDX spectra reveal the elemental composition of spherules in the killed and sterile-filtered controls, respectively.

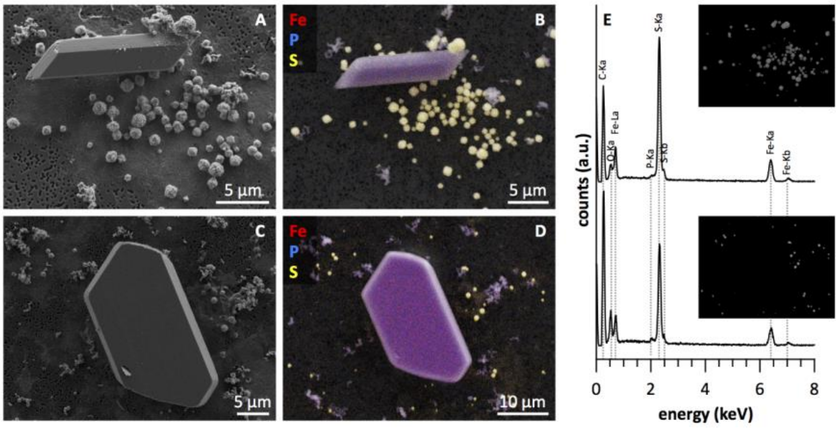

Figure SI4| SEM images of the 12-month old original enrichment culture, showing pyrite spherules (arrows) mixed with Fe-phosphates, including vivianite crystals (dotted arrows).

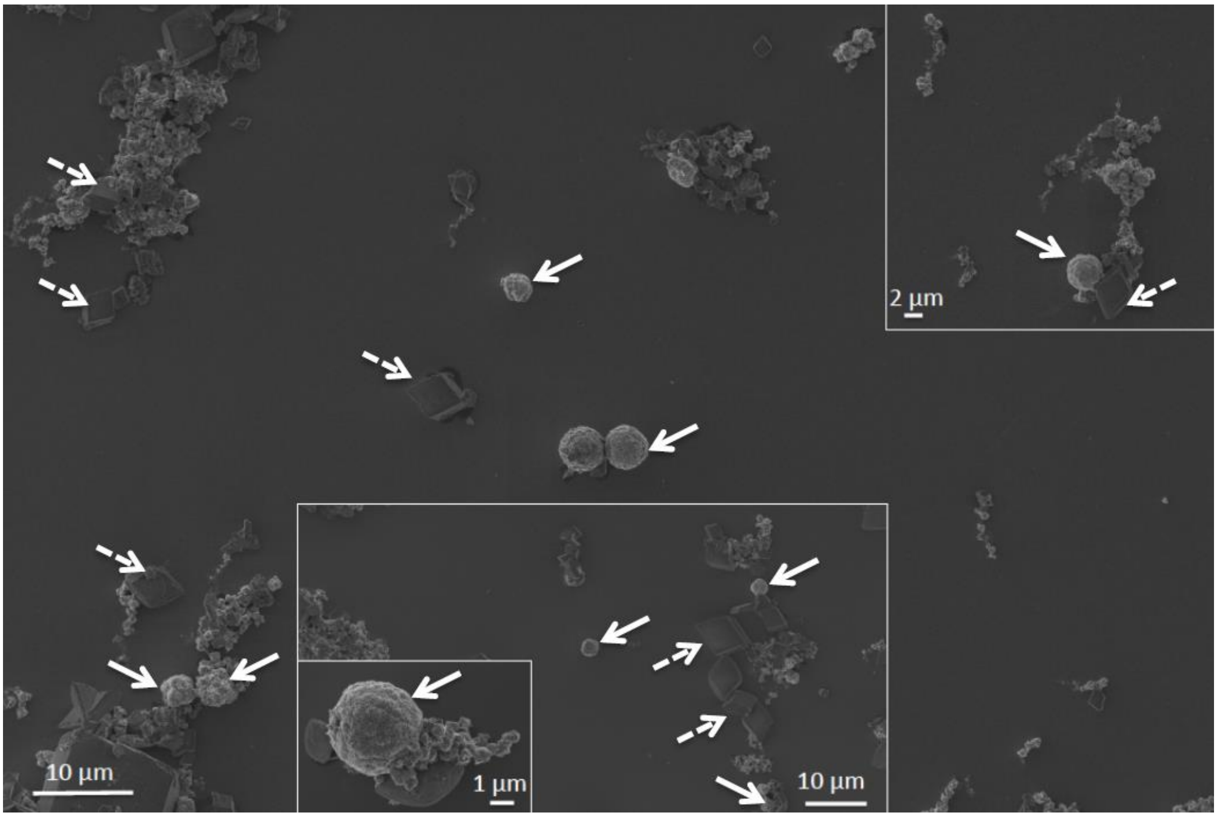

Figure SI5| Thermodynamic simulation (CHESS) of the evolution of dissolved and solid species concentrations depending on sulfide concentration. In this simulation, different concentrations of sulfide were added as dissolved Na_2S to a pH 6.3 medium containing 11 mM solid Fe(III)-PO_4.

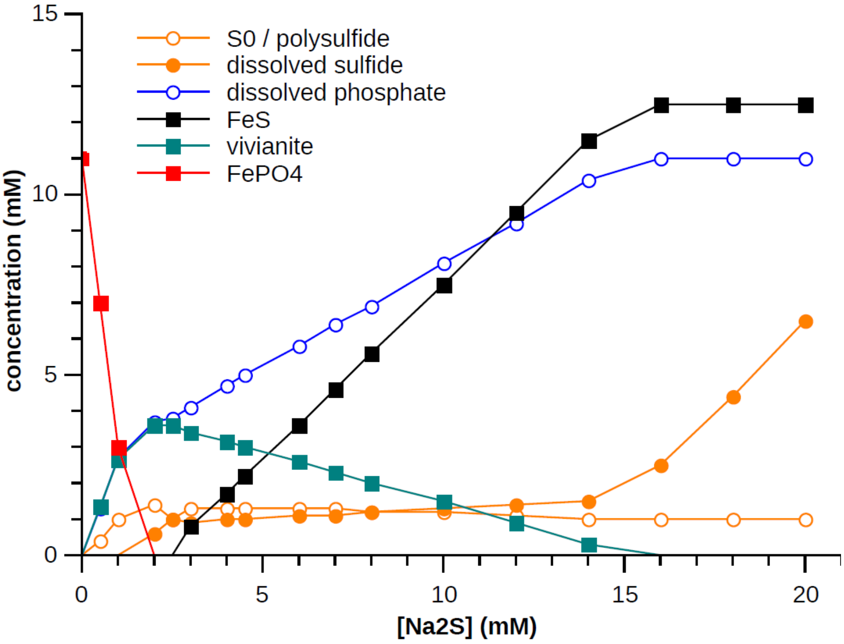

Table SI1| Mineralogy of Fe-bearing phases formed in the original enrichment culture from Lake Pavin water with added amorphous Fe(III)-phosphate, lactate and sulfate, after 75 days of incubation. Mineralogy and proportions of minerals were deduced from Fe K-edge $k_3\chi(k)$ EXAFS spectra Linear Combination Fits (LCF) (χ^2 of the fit = 2.5 $\cdot 10^{-2}$).

Fe mineral identity	Fe-mineral proportions
Vivianite	52 %
Fe(III)-phosphate	10 %
Mackinawite + Greigite	27%
Pyrite	13 %

REFERENCES

1. Wei, D. & Osseo-Asare, K. Aqueous synthesis of finely divided pyrite particles. *Colloids and Surfaces A: Physicochemical and Engineering Aspects* **121**, 27–36 (1997).
2. Noël, V. *et al.* EXAFS analysis of iron cycling in mangrove sediments downstream a lateritized ultramafic watershed (Vavouto Bay, New Caledonia). *Geochimica et Cosmochimica Acta* **136**, 211–228 (2014).
3. Chang, Y.-S., Savitha, S., Sadhasivam, S., Hsu, C.-K. & Lin, F.-H. Fabrication, characterization, and application of greigite nanoparticles for cancer hyperthermia. *Journal of Colloid and Interface Science* **363**, 314–319 (2011).
4. Donald, R. & Southam, G. Low temperature anaerobic bacterial diagenesis of ferrous monosulfide to pyrite. *Geochimica et Cosmochimica Acta* **63**, 2019–2023 (1999).
5. Miot, J. *et al.* Iron biomineralization by anaerobic neutrophilic iron-oxidizing bacteria. *Geochimica et Cosmochimica Acta* **73**, 696–711 (2009).

6. Mirvaux, B. *et al.* Iron Phosphate/Bacteria Composites as Precursors for Textured Electrode Materials with Enhanced Electrochemical Properties. *J. Electrochem. Soc.* **163**, A2139–A2148 (2016).
7. Ravel, B. & Newville, M. ATHENA, ARTEMIS, HEPHAESTUS: data analysis for X-ray absorption spectroscopy using IFEFFIT. *J Synchrotron Rad* **12**, 537–541 (2005).
8. Winterer, M. XAFS - A Data Analysis Program for Materials Science. *J. Phys. IV France* **7**, C2-243-C2-244 (1997).
9. Adra, A. *et al.* Arsenic Scavenging by Aluminum-Substituted Ferrihydrites in a Circumneutral pH River Impacted by Acid Mine Drainage. *Environ. Sci. Technol.* **47**, 12784–12792 (2013).
10. Miot, J. *et al.* Mineralogical Diversity in Lake Pavin: Connections with Water Column Chemistry and Biomineralization Processes. *Minerals* **6**, 24 (2016).
